# Sustainable utilization of bovine adipose tissue derivatives as robust antimicrobial agents against Methicillin-resistant *Staphylococcus aureus*

**DOI:** 10.1186/s12866-025-03747-5

**Published:** 2025-01-29

**Authors:** Muhammed Abdelhameed Ismael Alcici, Salma Waheed Abdelhaleem, Karima Mogahed Fahim, Neveen Mohamed Saleh, Heba Saeed Farag

**Affiliations:** 1 Department of Virology, Faculty of Veterinary Medicine, Cairo University, Giza, Egypt; 2 Department of Clinical Pathology, Faculty of Veterinary Medicine, Cairo University, Giza, Egypt; 3 Department of Food Hygiene and Control, Faculty of Veterinary Medicine, Cairo University, Giza, Egypt; 4 Egyptian Drug Authority (former National Organization for Drug Control and Research), University of Nebraska Medical Center (UNMC), Omaha, NE, USA; 5 Department of Internal Medicine and Infectious Diseases (Infectious Diseases), Faulty of Veterinary Medicine, Cairo University, Giza, Egypt

**Keywords:** MIMACs, Adipocytes, Adipolysate, Antimicrobial Resistance, MRSA, Sustainability, Fatty acid profile, GC/MS

## Abstract

**Background:**

The excessive use of antibiotics is a major contributor to the global issue of antimicrobial resistance (AMR), a significant threat to human and animal health. Hence, assessing new strategies for managing Multi-Drug Resistant (MDR) microorganisms is vital. In this study, the use of mechanically isolated mature adipose cells (MIMACs) and their lysate (Adipolysate) as a new sustainable antimicrobial agent was assessed against Methicillin-resistant *Staphylococcus aureus* (MRSA).

**Conclusions:**

The minimum volume of MIMACs achieved complete bacterial inhibition (Minimum Lethal volume) was 75 µl and 100 µl for bacterial concentration of 10^10^ and 10^12^ cfu/ml, respectively. Direct bacterial membrane attachment and intracellular capture was visualized under light and electron microscopy. Adipolysate was characterized via GC–MS, the fatty acid profile demonstrated several components with known antimicrobial properties. The tested Adipolysate revealed inhibition zone of diameter 25.33 ± 0.88 mm against the tested *S. aureus* strain, compared with the inhibition zone of Vancomycin (24.0 ± 0.00 mm) and Erythromycin (30.0 ± 0.00). The study revealed the potential effects of MIMACs and Adipolysate as sustainable, natural, and robust antimicrobial agents. However, these preliminary results will be further investigated to understand the mechanism of action and explore possible applications in various fields.

## Background

Undoubtedly, microbial evolution nowadays with rapid antimicrobial resistance (AMR) is one of the most daunting public health challenges, posing serious threats to humans, animals, and the environment [1]. AMR has been referred to as a "shadow pandemic" by researchers and a top public health threat by the WHO [2]. There were an estimated 4.95 million deaths associated with bacterial AMR in 2019, with *S. aureus* as the second most common causative agent associated with resistancecurrent evidence suggests the likelihood of dying from AMR is estimated to be more than 1.5 times higher for individuals in low- and middle-income countries compared to those in wealthier countries, raising the need for sustainable and economical solutions to AMR [3].

Many systemic and local infections are caused by Methicillin-resistant *Staphylococcus aureus* (MRSA), include skin and soft tissue infections (like abscesses, boils, and cellulitis), pneumonia, bloodstream infections (bacteremia and sepsis), bone and joint infections (osteomyelitis and septic arthritis), surgical site infections, urinary tract infections, toxic shock syndrome, necrotizing fasciitis, and mastitis, which is known to become resistant to routinely provided medications quickly. As a result, the range of incurable MRSA infections is growing by the emergence of new, highly resistant strains, such as USA300 (community-acquired MRSA) and USA100/200 (hospital-acquired MRSA), which have developed resistance to multiple antibiotics, including vancomycin (VRSA), linezolid, and daptomycin. These strains use various resistance mechanisms, such as altered target sites, efflux pumps, enzymatic modification, and biofilm formation, which hinder the effectiveness of last-line antibiotics and complicating treatment.

In response, researchers are exploring new antibiotics like ceftaroline and oritavancin, combination therapies, immunotherapies, and phage therapy to combat these resistant strains. However, the rising resistance to existing treatments highlights the urgent need for innovative, sustainable, and economical methods to combat the growing public health threat of AMR and cure MRSA infections [4].

Current antimicrobial alternatives include phage therapy, quorum sensing targeted therapy, nanoparticle-based therapy, and antimicrobial peptides. Some of these substitutes are not yet applicable due to considerable impediments: high expense and small-scale production that limits their market availability, resistance, narrow activity spectrum, chemical stability, and determination of working concentrations [4]. However, cell-based therapeutics are an emerging modality with the potential to treat many complex diseases through distinct modes of action [5].

Adipose tissue therapy has been widely used in regenerative medicine [6, 7]. This was partly due to the discovery of adipose-derived stem cells (ADSC) and stromal vascular fraction (SVF) and their roles in angiogenesis and immunomodulation [8]. Adipocytes, previously perceived as mere energy stores, have proven to play critical immunological roles mainly through the action of adipokines. Adipokines are a group of bioactive factors produced by adipocytes; they include over 600 cytokines and hormones [9, 10] that activate or suppress immune responses both systemically and within adipose tissue [11]. Nonetheless, evidence of direct immunological functions for adipocytes has been slight, but recent studies have identified the role of subcutaneous adipocytes in barrier immunity as a significant source of antimicrobial peptides (AMPs), which can directly kill bacteria [12]. In addition, fatty acids are thought to be a sustainable therapy option for infectious disorders in place of traditional antibiotics owing to their antibacterial, anti-virulence, and anti-biofilm characteristics [13, 14].

**Fig. 1:**
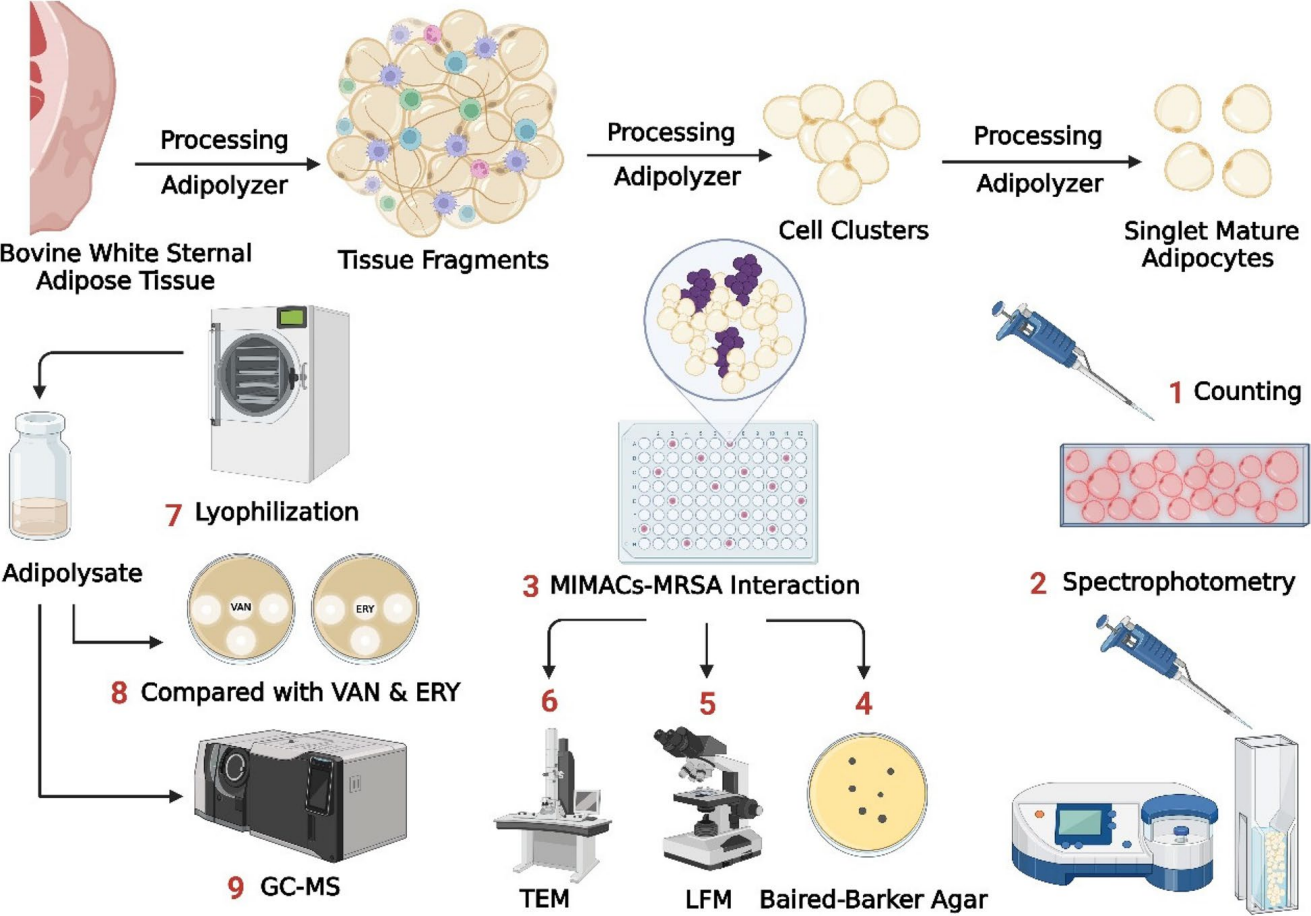
A flowchart showing all the methods described below under agreement number *PZ27J7GZW8* Created in BioRender https://biorender.com/d05w466

The present study demonstrates a new, simple, and economical technique that aims to use mechanically isolated mature adipocytes (MIMACs) and their lysate (Adipolysate) to explore their potent antimicrobial effect against methicillin-resistant *S. aureus* (MRSA) as well as capture the interaction between MIMACs and *S. aureus*, in vitro Fig. 1.

## Material and methods

### Sample collection and processing

White sternal subcutaneous adipose tissue samples of ten Holstein bulls were aseptically collected after Islamic slaughter from El-Bassatin automated slaughterhouse in Cairo, Egypt. Samples were kept in opaque glass bottles with 0.9% normal saline during transportation. Bulls' age and body weight are described as mean ± SEM (2.3 ± 0.6 years; 416.6 ± 25.5 kg).

### MIMACs preparation

**Fig. 2:**
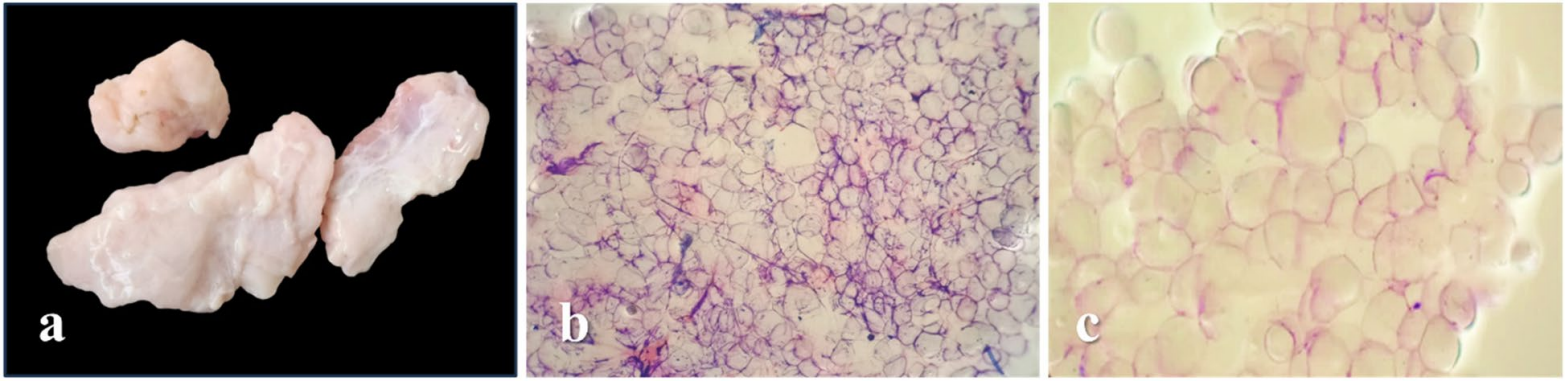
MIMACs preparation **a** fragments of adipose tissue prepared for Adipolyzer processing. **b** adipocyte clumps yielded after initial processing. **c** MIMACs, after secondary processing at 1200 rpm with multiple filtrations and washing procedures, appear as individual cells

The collected samples were rinsed with 0.9% normal saline and trimmed with sterile scissors to remove any obvious attached surrounding fascia and vessels. Then 50 g of each sample was dissected to produce fragments of approximately 25 – 30 mm^3^ (Fig. 2-a). After that, the tissue fragments were transferred to the Adipolyzer (Egyptian patent office with application number EG/P/2024/228) with 200 ml of neutral solution (pH 7.1) to make tissue suspension that was mechanically digested at 1800 rpm and passed through a 150-μm mesh filter (Fig. 2-b).

A filtered suspension was further centrifuged at 1200 rpm for 5 min, and the formed white upper layer (MIMACs) was transferred to a 20 ml collecting syringe, washed with 0.9% normal saline three times, and then used freshly (Fig. 2-c).

### MIMACs count estimation

After processing, for every 50 g of bovine adipose tissue, 18 ± 0.37 ml of MIMACs were yielded. MIMACs were diluted 1.5 times with 0.9% normal saline and prepared in incremental volumes (50 µL,100 µL,150 µL, and 200 µL) for cell counting using image processing software (Fiji—ImageJ). The same volumes were loaded into flat-bottom, 96-well microtiter plates and spectrophotometrically measured to determine their optical densities (OD) at 600 nm (FLUOstarOmega, BMG Labtech, Germany); the process was repeated in triplicates.

### Adipolysate preparation

The adipocytes were mixed with 0.9% normal saline (V/V) and lyophilized using a freeze dryer (BUCHI Lyovapor L-200). The dried lyophilized Product was kept in a sealed glass container at -20 °C in the dark until use.

### Preparation of MRSA strain

The pure culture of methicillin-resistant *S. aureus* 43,300 (MRSA) reference strain was grown overnight in Brain Heart Infusion broth (Hi-Media) containing 0.3% yeast extract at 37 °C. The bacterial concentrations of 10^8^, 10^10^, and 10^12^ CFU/ml were prepared by serial dilution and surface spreading on duplicate plates of Baird-Parker agar medium (Hi-Media) supplemented with egg yolk potassium tellurite [15].

### Assessing of MIMACs and Adipolysate antimicrobial activity against MRSA

MIMACs 100 µl from the prepared MRSA culture in different concentrations, different bacterial loads C1 (10^10^) and C2 (10^12^) were incubated directly with adipocytes volumes of 50, 100, 150, and 200 µl, in sterile tubes separately, at 37 °C for 24 h. 100 µl from the incubated mixture was spread into duplicate plates of Baird-Parker media and incubated at 37 °C for 24 h. Control plates were prepared by spreading 100 µl from the pure bacterial culture.

The microbial reduction percentage was calculated as follows:

$$\begin{array}{c}(\mathrm{Control}\;\mathrm{CFU}-\;\mathrm{Test}\;\mathrm{CFU})\;/\;\mathrm{Control}\;\mathrm{CFU})\;\mathrm x\;100\\\mathrm{CFU}:\;\mathrm{Colony}\;\mathrm{Forming}\;\mathrm{Unit}\end{array}$$

#### Adipolysate

Pure bacterial culture was standardized using McFarland's standard 0.5, then inoculated into Mueller–Hinton agar plates using a sterile cotton swab. Agar wells were punched in the plates and then filled with 100 µg Adipolysate, standard antibiotic discs of 15 µg Erythromycin (Oxoid Ltd., Basingstoke England), and 30 µg Vancomycin (Bioanalyse, Ankara, Türkiye) were placed onto the plates. The plates were incubated at 37 °C for 24 h and examined for the zone of inhibition. The experiment was carried out in triplicates.

### Detection of MIMACs and MRSA interaction by bright-field and TEM

Equal volumes of the used MRSA culture and MIMACs were incubated at 37 °C for 24 h, and cytologic smears were prepared from the suspension mixture, stained with eosin and methylene blue (Merck, Germany), and examined under bright-field microscopy (BX50F4, Olympus, Tokyo, Japan). The remaining incubation suspension was centrifuged at 3000 × g for 15 min., and the precipitate was collected for examination by TEM (JEM1400, JEOL, USA) at the TEM unit of the Research Laboratories' Complex, Faculty of Agriculture, Cairo University, Egypt.

### Fatty acid profile of Adipolysate using GC/MS

The ISO 12966–2:2017 rapid method was used for preparing fatty acid methyl ester (FAME) [16]. The test portion of about 100 mg of bovine Adipolysate was introduced into a 10 ml screw top test tube with 2 ml of isooctane and 0.1 ml of potassium hydroxide (2N), and the mixture was vortexed till turbidity appeared, then it was allowed to stand for 2 min, 2 ml of sodium chloride (40%) were added and vortexed for one minute. The two phases were separated, and the upper layer was drawn out and mixed with 1 g of anhydrous sodium hydrogen sulfate. Finally, 2 ml of isooctane was added, and the mixture filtered with the upper isooctane layer was transferred to a sample vial and injected directly into a Thermo Scientific Trace GC Ultra / ISQ Single Quadrupole MS, TG-5MS fused silica capillary column (30 m, 0.251 mm, 0.1 mm film thickness) for the GC/MS analysis. Helium gas was employed as the carrier gas at a steady flow rate of 1 mL/min in an electron ionization system with an ionization energy of 70 eV for GC/MS detection. The temperature of the MS transfer line and injector was fixed at 280 °C.

The oven temperature was set to start at 40 °C (hold 3 min) and increase by 5 °C each minute (hold 5 min) to 280 °C as the ultimate temperature. Using a percent relative peak area, the quantification of each detected component was examined. Comparing the compounds' relative retention times and mass spectra with the GC/MS system's NIST, WILLY library data allowed for a provisional identification of the substances. All analyses were performed in triplicate.

### Statistical analysis

The obtained data were statistically analyzed using the software program SPSS version 17.0 (IBM, New York, USA). The results were expressed as Mean ± SEM. Adipolysate and antibiotics inhibition zone results were compared using a *t*-test, where the differences were significant at *p*-value < 0.05.

## Results

### Antimicrobial activity of MIMACs on MRSA

**Fig. 3:**
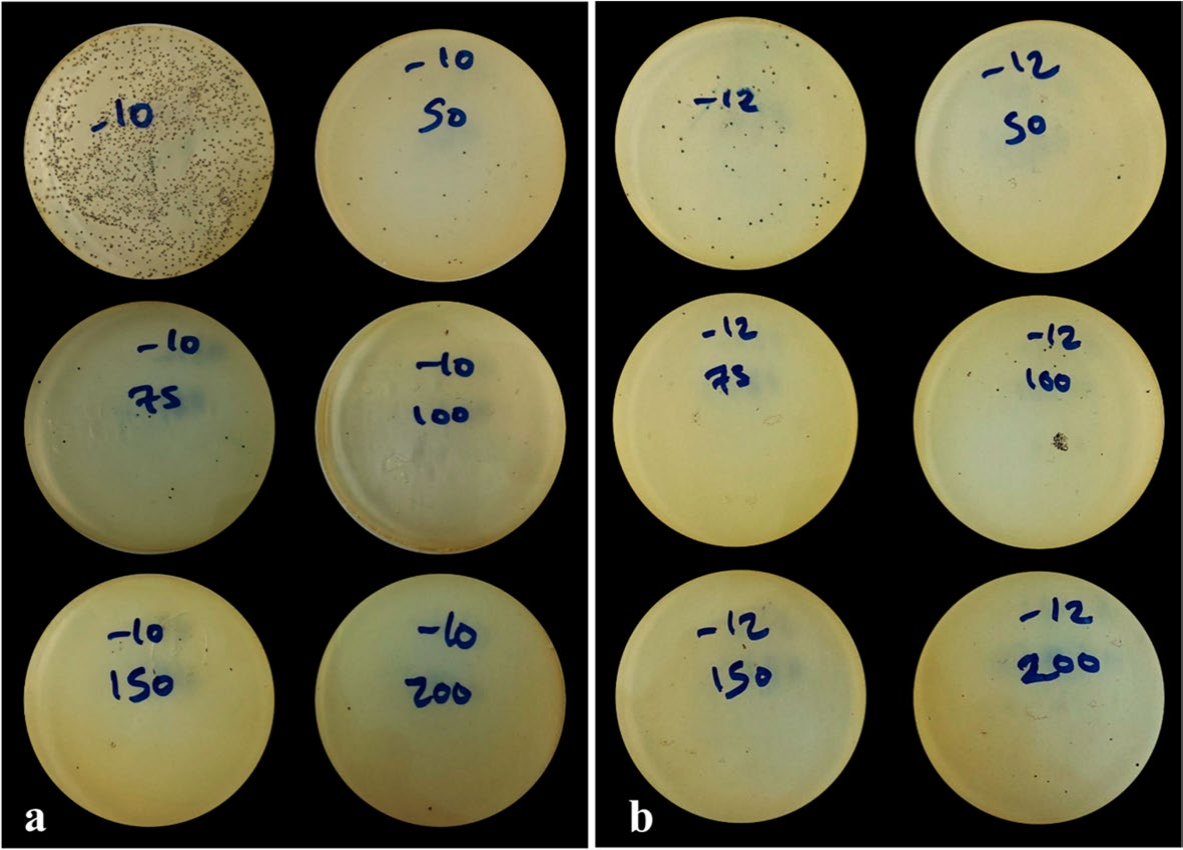
The antimicrobial effect of MIMACs against MRSA on Baird Parker agar plates. **a** bacterial count 10^10^, **b** bacterial count 10.^12^

The estimated cell counts and OD600 readings corresponded accordingly with the incremental cell suspension volumes; the higher the volume, the higher the cell count and OD600 readings (Table 1). The reduction of bacterial growth increased with increasing volumes of MIMACs; the minimum volume of MIMACs achieved complete bacterial inhibition (Minimum Lethal volume) was 100 µl and 75 µl for bacterial concentrations of 10^10^ and 10^12^ cfu/ml, respectively, shown in (Table 2) and (Fig. 3).

**Table 1:** Estimated cell counts and OD600 of MIMACs. Results are presented as means ± standard error of means (SEM)

Volumes	Cell count means	SEM	OD600 means	SEM
50 µL	559 cells	44.37	0.78	0.23
100 µL	855 cells	32.82	1.86	0.15
150 µL	975 cells	35.02	2.37	0.17
200 µL	1251 cells	75.78	2.92	0.20

**Table 2:** Antimicrobial activity and reduction rate of MIMACs on MRSA growth

MIMACs Volumes	Bacterial count	Reduction%	Bacterial count	Reduction%
	**C**_**1**_ (10^10^) 89.66 × 10^10^ ± 8.3910^9^		**C**_**2**_ (10^12^) 44.00 × 10^12^ ± 20.81 × 10^11^	
**50 µl MIMACs**	17.33 × 10^10^ ± 14.52 × 10^9^	80.67	66.66 × 10^10^ ± 28.48 × 10^10^	98.49
**75 µl MIMACs**	80.0 × 10^9^ ± 15.27 × 10^9^	91.08	0.0	100
**100 µl MIMACs**	0.0	100	0.0	100
**150 µl MIMACs**	0.0	100	0.0	100
**200 µl MIMACs**	0.0	100	0.0	100

#### Adipolysate sensitivity testing

**Fig. 4:**
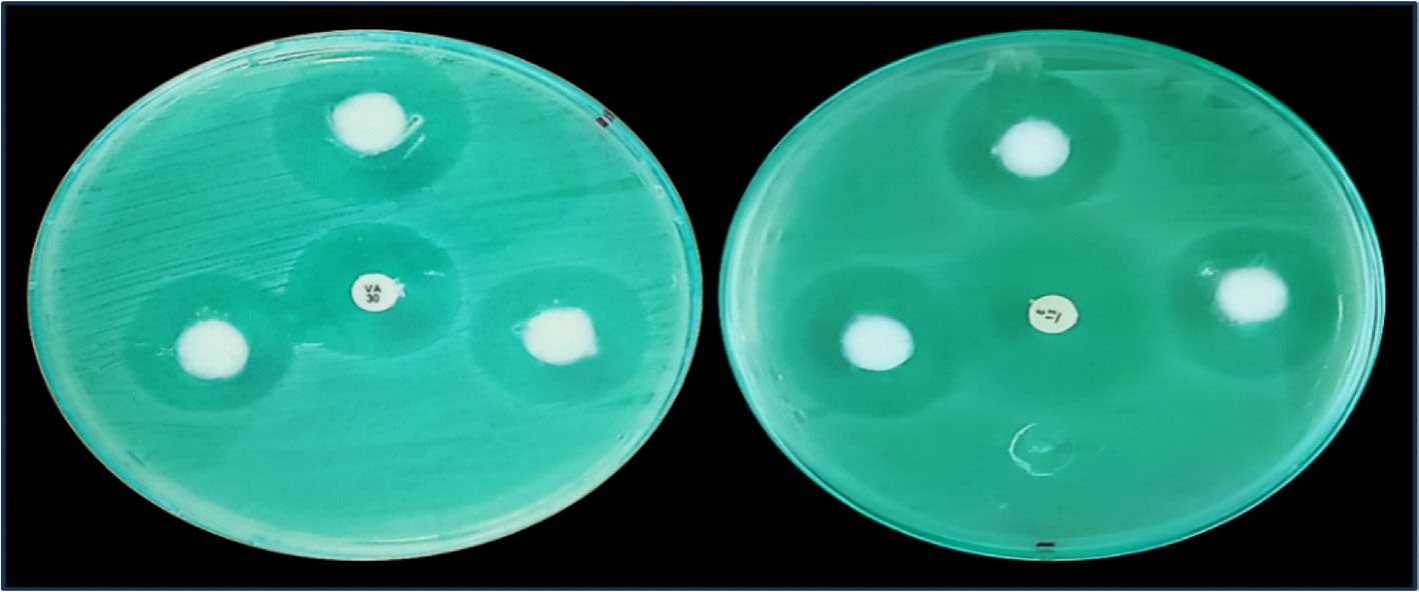
Antimicrobial Sensitivity of Adipolysate compared with vancomycin (left) and erythromycin (right) on Muller Hinton agar plates

The tested Adipolysate revealed an inhibition zone of diameter 25.33 ± 0.88 mm against the tested MRSA strain, compared with the inhibition zone of vancomycin (24.0 ± 0.00 mm) showing non-significant difference (t (2.0) = -1.512, *p* = 0.270). At the same time, Adipolysate showed an inhibition zone of diameter 25.67 ± 0.88 mm compared with the inhibition zone of erythromycin (30.0 ± 0.00), with a significant difference (*t* (2.0) = 4.914, *p* = 0.039), shown in (Table 3) and (Fig. 4).

**Table 3:** Antimicrobial Sensitivity of Adipolysate compared with vancomycin and erythromycin

	Inhibition zone (mm) Mean ± SEM	*p*-value
**Vancomycin**	24.0 ± 0.00	0.270
**Adipolysate**	25.53 ± 0.88	
**Erythromycin**	30.0 ± 0.00	0.039*
**Adipolysate**	25.67 ± 0.88	

^*^ The difference was significant at *p* < 0.05

#### Detection of MIMACs and MRSA interaction by bright-field and TEM

**Fig. 5:**
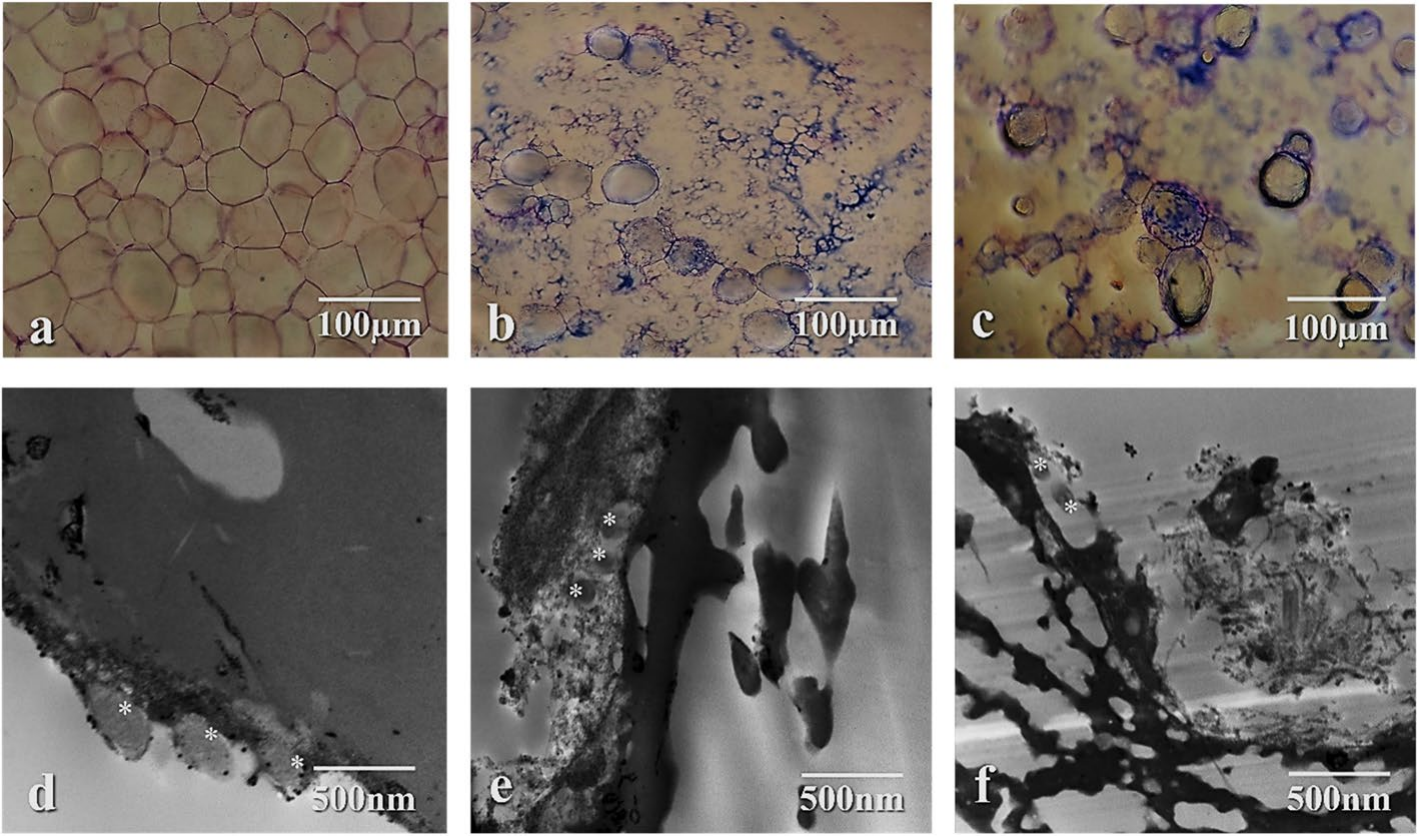
Photomicrographs displaying the MRSA-MIMACs interaction. **a**-**c** images by bright-field microscope, **d**-**f** images by TEM (asterisks indicate the *S. aureus* particles)

The bright-field photomicrographs showcase the difference in adipocyte morphology between the control negative MIMAC smear, where healthy typical mature adipocytes can be seen in close contact with one another (Fig. 5-a), compared to smears prepared from the incubated suspension of MIMACs with MRSA, as the aftermath of their interaction is displayed in adipocyte membrane lysis (Fig. 5-b) and the presence of intracellular bacteria (Fig. 5-c). TEM photomicrographs revealed in further detail the bacterial attachment to the adipocyte surface (Fig. 5-d), the intramembrane bacterial capture (Fig. 5-e), and the damaged adipocyte membrane (Fig. 5-f).

#### Fatty acid profile of Adipolysate using GC/MS

GC/MS analysis revealed the identification of ten compounds displayed in (Table 4), where 9-octadecenoic acid (Z)—methyl ester, hexadecenoic acid- methyl ester, and methyl stearate are represented as the major compounds with 40% and 28%, and 19%, respectively of the total identified compounds. The result showed that the saturated fatty acids represented the vast majority (52.9%), while the unsaturated fatty acids constituted (47.1%) of the total identified compounds.

**Table 4:** Fatty acid profile of bovine Adipolysate

	Fatty acid	AreaSum(%)	CAS ID	R_T_ (min.)
1	Methyl tetradecanoate	5	124–10-7	26.428
2	Methyl myristoleate^a^	1	56,219–06-8	27.335
3	Pentadecanoic acid, methyl ester	0.5	7132–64-1	28.477
4	Hexadecanoic acid, methyl ester	28	112–39-0	30.576
5	Palmitoleic acid^a^	4	1120–25-8	31.05
6	Heptadecanoic acid, methyl ester	0.4	1731–92-6	31.532
7	Methyl stearate	19	112–61-8	34.414
8	9-Octadecenoic acid (Z)-, methyl ester^a^	40	112–62-9	34.684
9	9,12-Octadecadienoic acid, methyl ester^a^	1.1	2462–85-3	36.352
10	cis-13,16-Docasadienoic acid^a^	1.0	7370–49-2	46.531
Total saturated		52.9%		
Total unsaturated		47.1%		

^a^Refers to unsaturated FA

## Discussion

Antimicrobial resistance poses a major threat to human and animal health worldwide [2, 3]. Many treatments are currently under exploration, but cell-based therapeutics show a promising, sustainable antimicrobial alternative [17].

For the longest time, adipose tissue was considered an inert physical barrier playing a role in metabolism and heat insulation [18], and when the therapeutic effects were investigated, most studies focused on adipose-derived stem cells and the stromal vascular fraction rather than the adipocyte fraction [8, 19–21].

The most common method of adipose tissue processing depends on using enzymatic digestion (collagenase and trypsin). However, enzymatic isolation techniques are quite costly and time-consuming; this led to the consideration of mechanical tissue dispersion methods using shear force, centrifugation, or pressure [22–24]. The work describes the use of the Adipolyzer (under review at the Egyptian Patent Office – application number EG/P/2024/228), a mechanical adipocyte isolation system that performs in a reproducible, timely, and cost-efficient manner. The isolated adipocyte quantity was estimated by cell count and OD measurement at 600 nm, given that OD600 has long been used as a method of cell count estimation [25–27]. The present study showed a correspondence between both techniques, enabling the use of OD600 instead of the more time-consuming procedure of cell counting; this should facilitate future MIMACs' count estimation.

Subcutaneous adipose tissue was selected to be used in this study for its high content of connective tissue, saturated and unsaturated fatty acids, as well as its semisolid state at room temperature being quickly processed and getting high yield, compared to perirenal adipose tissue that has low connective tissue, high unsaturated fatty acids, solid in room temperature being hardly processed with low yield [28].

Evidence of direct immunological functions for adipocytes has been slight and mostly limited to specialized adipose tissue proximal to lymph nodes known as perinodal adipose tissue (PAT). PAT was thought to provide a readily available supply of fatty acids to activate lymphocytes, preventing them from competing for blood nutrients and thus improving immune responses [29]. Interestingly, subcutaneous inoculation of bacteria resulted in infection of PAT, and these adipocytes were able to clear intracellular bacteria by functional refocusing towards an immune response by upregulating genes associated with host defense and downregulating those related to fat metabolism [30].

Previous work has demonstrated that a crucial host defense mechanism against skin infections is a localized rise in subcutaneous adipocytes, size and number, after a *S. aureus* subdermal infection in mice; this finding demonstrates that adipocytes create an antimicrobial peptide cathelicidin, which has been shown to suppress the development of bacteria, activate neutrophils, and have pro-inflammatory properties [10, 31]. Additionally, adipocytes can exert immunological effects by sharing common antigen receptors with macrophages and by secretion of pro-inflammatory cytokines and chemokines such as TNF and IL-6 upon stimulation, as well as the production of adipokines and AMPs [11, 12, 29, 32].

The current study focused on the antibacterial role of adipocytes and Adipolysate on one of the most common opportunistic pathogens and commensal bacteria, *S. aureus.* It can cause potentially fatal diseases and a variety of illnesses, such as skin infections, pneumonia, and sepsis. Moreover, *S. aureus* was the most common cause of contagious mastitis in the bovine dairy herd; according to the survey study conducted on 20 randomly selected Egyptian dairies, contagious mastitis was reported on 45.2% of the study dairies, specifically *S. aureus* (35.7%) [33].

The study findings demonstrated that MIMACs could inhibit the growth of MRSA in vitro, with a Minimum Lethal volume of 75 µl and 100 µl for bacterial concentration of 10^10^ and 10^12^ cfu/ml. The extract Adipolysate showed a high antibacterial effect on MRSA, which was close to the results of the antibiotics used, vancomycin and erythromycin. These findings support the potential use of MIMACs and their Adipolysate as a safe, effective, and sustainable antimicrobial alternative.

Arguably, this is the first documented interaction between *S. aureus* and adipocytes by bright-field and transmission electron microscopy; the photomicrographs display the membrane attachment, disruption, and subsequent invasion of *S. aureus* into MIMACs, which supported the results of the in vitro antibacterial activity. *S. aureus* can infect adipocytes and remain viable within these cells; in fact, *S. aureus* infection of adipocytes results in decreased release of adiponectin and resistin, while secretion of IL-6, visfatin, and monocyte chemoattractant protein-1 (MCP-1) is enhanced.

There has been speculation that adipose tissue and the adipocytes within them could serve as hosts for a persistent *S. aureus* infection because adipocyte viability is unaffected by infection [10]. The presented results implied adipocytes' engulfment and bactericidal effects after contact with the bacterial cells that parallel previous findings, whichdemonstrated the wide range of antibacterial activities of adipose-derived mesenchymal stromal cells (ASCs) against both gram-positive and gram-negative bacteria (*L. casei*, *S. aureus*, and *E. coli*, *En. Faecalis)*. After six hours of contact, ASCs have a bactericide-like effect through oxygenated free radical release, phagocytosis, and antibacterial molecule production. Following ASCs contact with *S. aureus* cells, abnormalities in *S. aureus* division resulted in pseudo-multicellular structures. These findings show that contact is crucial for the antibacterial actions of ASCs, which reduce bacterial growth and membrane permeabilization [20].

Some fatty acids have antibacterial properties by directly inhibiting or killing bacteria, while others have an impact on virulence factors or stop bacteria from adhering to surfaces; these effects depend on their structures, morphologies, carbon chain length, and the quantity, locations, and orientation of double bonds [13, 14]. Many organisms use fatty acid methyl ester's antibacterial qualities to defend against bacteria. Its primary action target is the bacterial cell membrane; it obstructs the synthesis of cellular energy, inhibits the functioning of enzymes, and ultimately causes the direct lysis of bacterial cells.

Additionally, fatty acids inhibit biofilm formation and lower hyphae or fimbriae production [34, 35]. Thus, the fatty acid methyl ester is a potentially effective antimicrobial drug due to its safety and efficacy [36]. The fatty acid profile of bovine Adipolysate represented Methyl tetradecanoate, Methyl myristoleate, Pentadecanoic acid, Hexadecanoic acid, Palmitoleic acid, Heptadecanoic acid, Methyl stearate, Octadecenoic acid, Octadecadienoic acid and Docasadienoic acid as the main components. Several studies demonstrated the antimicrobial activity of different fatty acids, such as tetra-decanoic acid and pentadecanoic acid, which were reported to cause significant inhibition and disruption of *E. coli*, *En. Faecalis*, and *S. enterica* serovar Typhimurium [37–39]. Tetradecanoic acid (myristic acid) alleviates a number of *C. albicans* virulence pathways, including oxidative stress mitigation, sphingolipid metabolism, and ergosterol production [40].

Additionally, the presented findings were analogous to the results of other conducted studies that analyzed the chemical compounds of *Imperata cylindrica and Scenedesmus intermedius* using GC/MS analysis. Certain substances examined, including hexadecanoic acid methyl ester, were discovered to possess antibacterial influence against gram-positive and gram-negative bacteria such as *S. aureus, P. aeruginosa, B. subtilis, and K.* [41, 42].

Myristoleic acid inhibited the formation of *S. aureus* biofilm, as it influences the expression of various genes relevant to biofilm formation, including virulence-related genes, lipase, and hyaluronate lyase [43]. 9,12-octadecadienoic acid was among the fatty acid esters with antimicrobial activity against *S. aureus*, *S. epidermidis*, *B. subtilis*, *E. coli,* and *Ps. Aeruginosa* [44], while another work showed 9,12-octadecadienoic acid and hexadecanoic acid possess significant inhibitory activity against *S. aureus* and *B. subtilis* [45]. The antibacterial effect against gram-positive and gram-negative bacteria was attributed to several fatty acids, including 9-octadecenoic acid, methyl ester, and heptadecanoic acid [46].

Furthermore, several fatty acids exhibited diverse biological properties; tridecanoic acid (TDA) has anthelminthic, anti-inflammatory, and anti-cancer effects [47]. Pentadecanoic acid shows promise in augmenting the effectiveness of endocrine therapy for the treatment of breast cancer cells [48]. Monounsaturated hexadecenoic fatty acids have been given more attention in recent years as potential health indicators because of their essential roles in physiology and pathology. According to some descriptions, palmitoleic acid (an isomer of hexadecenoic acid) is a lipokine that can control a variety of metabolic processes, including lipogenic activity in white adipocytes, β cell proliferation, muscle insulin sensitivity, and endoplasmic reticulum stress prevention. Palmitoleic acid has been linked to several advantageous effects in rodent models and cell lines [49].

Antimicrobial resistance results in *S. aureus* treatment failure, this raises an urgent need to limit traditional treatment with antibiotics and to explore new alternatives of combat [50]. This study aimed to provide such an alternative, but it had some limitations including a small sample size, which may affect the generalizability of the results and variability in antimicrobial properties. Antimicrobial effects were assessed in vitro, potentially overlooking the complexities of in vivo conditions, and the mechanisms behind the observed activity were not thoroughly investigated. While comparisons were made with certain antibiotics, a broader range of antimicrobials and multidrug-resistant pathogens should be included in future research. The need to evaluate the cytotoxicity of MIMACs and Adipolysate remains unaddressed, along with the implications of storage conditions on their efficacy over time.

The research offers novel insights into the potential of bovine adipose tissue derivatives, specifically mechanically isolated mature adipose cells (MIMACs) and their lysate (Adipolysate), as sustainable and effective antimicrobial agents against methicillin-resistant *Staphylococcus aureus* (MRSA). The study demonstrates significant antimicrobial activity, with complete bacterial inhibition observed at specific volumes, suggesting that these adipose-derived products could serve as alternatives to traditional antibiotics, thus addressing the critical issue of antimicrobial resistance. Additionally, the characterization of the fatty acid profile through GC/MS spectrometry highlights specific components with known antimicrobial properties, paving the way for further exploration into their mechanisms of action and potential applications in medical and agricultural fields.

## Conclusion

There is no well-known antimicrobial agent to support the successful management of the multi-drug resistance (MDR) crisis despite a significant growth in pioneering technologies to prevent the establishment of MDR. As a result, research attention has switched to non-conventional antimicrobial formulations to reduce the MDR epidemic. The study revealed the antimicrobial effect of adipocytes and their Adipolysate against methicillin-resistant *Staphylococcus aureus* (MRSA). The fatty acid profile demonstrated several components with known antimicrobial properties. Thus, due to their efficacy and sustainability, MIMACs and Adipolysate are potentially applicable natural antimicrobial agents. However, these antimicrobial effects are preliminary and will be further investigated to understand the mechanism of action and explore possible applications in various fields.

## Data Availability

The datasets used and/or analyzed during the current study are available from the corresponding author upon reasonable request.

## Abbreviations

MDR: Multi-Drug Resistant
MIMACs: Mechanically isolated mature adipose cells
MRSA: Methicillin-resistant *Staphylococcus aureus*
GC/MS: Gas Chromatography/Mass spectrophotometry
AMR: Antimicrobial resistance
WHO: World Health Organization
*S.**aureus*: *Staphylococcus* *aureus*
PAT: Perinodal adipose tissue
AMPs: Antimicrobial peptides
OD: Optical densities
CFU/ml: Colony forming unit/milliliter
FAME: Fatty acid methyl ester
TEM: Transmission electron microscope
AMPs: Antimicrobial products

## References

[1] Zhao C, Wang Y, Mulchandani R, Van Boeckel TP. Global surveillance of antimicrobial resistance in food animals using priority drugs maps. Nat Commun. 2024;15. 10.1038/s41467-024-45111-7 PMC10817973 38278814

[2] WHO. Antimicrobial Resistance Fact Sheet. https://www.who.int/news-room/fact-sheets/detail/antimicrobial-resistance. Accessed 21 Nov 2023.

[3] Murray CJ, Ikuta KS, Sharara F, Swetschinski L, Robles Aguilar G, Gray A, Han C, Bisignano C, Rao P, Wool E, Johnson SC, Browne AJ, Chipeta MG, Fell F, Hackett S, Haines-Woodhouse G, KashefHamadani BH, Kumaran EAP, McManigal B, Naghavi M. Global burden of bacterial antimicrobial resistance in 2019: a systematic analysis. Lancet. 2022;399:10325. 10.1016/S0140-6736(21)02724-0 PMC8841637 35065702

[4] Cong Y, Yang S, Rao X. Vancomycin resistant *Staphylococcus aureus* infections: A review of case updating and clinical features. J Adv Res. 2019;12:21. 10.1016/j.jare.2019.10.005 PMC7015472 32071785

[5] Łojewska E, Sakowicz T. An alternative to antibiotics: selected methods to combat zoonotic foodborne bacterial infections. Curr Microbiol. 2021;78:12. 10.1007/s00284-021-02665-9 PMC8595143 34626217

[6] Ghiasloo M, Lobato RC, Díaz JM, Singh K, Verpaele A, Tonnard P. Expanding clinical indications of mechanically isolated stromal vascular fraction: A systematic review. Aesthetic Surg J. 2020;40:9. 10.1093/asj/sjaa111 32358957

[7] Busato A, De Francesco F, Biswas R, Mannucci S, Conti G, Fracasso G, Conti A, Riccio V, Riccio M, Sbarbati A. Simple and rapid non-enzymatic procedure allows the isolation of structurally preserved connective tissue micro-fragments enriched with SVF. Cells. 2021;10:1. 10.3390/cells10010036 PMC7824313 33383682

[8] Guo J, Nguyen A, Banyard DA, Fadavi D, Toranto JD, Wirth GA, Paydar KZ, Evans GRD, Widgerow AD. Stromal vascular fraction: A regenerative reality? Part 2: Mechanisms of regenerative action. J Plast Reconstr Aesth. 2016;69:2. 10.1016/j.bjps.2015.10.014 26546112

[9] Kirichenko TV, Markina YV, Bogatyreva AI, Tolstik TV, Varaeva YR, Starodubova AV. The role of adipokines in inflammatory mechanisms of obesity. Int J Mol Sci. 2022;23:23. 10.3390/ijms232314982 PMC9740598 36499312

[10] Barthelemy J, Bogard G, Wolowczuk I. Beyond energy balance regulation: The underestimated role of adipose tissues in host defense against pathogens. Front Immunol. 2023;14:1083191. 10.3389/fimmu.2023.1083191 PMC10019896 36936928

[11] Bradley D, Xu A, Hsueh WA. The Immunomodulatory Roles of Adipocytes. Front Immunol. 2021;12:827281. 10.3389/fimmu.2021.827281 PMC8732371 35003144

[12] Guan J, Wu C, He Y, Lu F. Skin-associated adipocytes in skin barrier immunity: A mini-review. Front Immunol. 2023;14:1116548. 10.3389/fimmu.2023.1116548 PMC9902365 36761769

[13] Olsen I. Biofilm-specific antibiotic tolerance and resistance. Eur J Clin Microbiol Infect Dis. 2015;34(5):877–86. 10.1007/s10096-015-2323-z 25630538

[14] Yoon BK, Jackman JA, Valle-González ER, Cho NJ. Antibacterial free fatty acids and monoglycerides: biological activities, experimental testing, and therapeutic applications. Int J Mol Sci. 2018;19(4):1114. 10.3390/ijms19041114 PMC5979495 29642500

[15] Salfinger Y, Tortorello ML. Compendium of methods for the microbiological examination of foods. 5th ed. Washington DC: APHA press; 2015.

[16] ISO 12966–2:2017, Animal and vegetable fats and oils — Gas chromatography of fatty acid methyl esters. Part 2: Preparation of methyl esters of fatty acid. 2^nd^ ed. 2017

[17] Bashor CJ, Hilton IB, Bandukwala H, Smith DM, Veiseh O. Engineering the next generation of cell-based therapeutics. Nat Rev Drug Discov. 2022;21:9. 10.1038/s41573-022-00476-6 PMC9149674 35637318

[18] Zwick RK, Guerrero-Juarez CF, Horsley V, Plikus MV. Anatomical, Physiological, and Functional Diversity of Adipose Tissue. Cell Metab. 2018;27(1):68–83 (**Cell Press**). 29320711 10.1016/j.cmet.2017.12.002 PMC6050204

[19] Aronowitz JA, Lockhart RA, Hakakian CS. Mechanical versus enzymatic isolation of stromal vascular fraction cells from adipose tissue. Springer Plus. 2015;4:1. 26636001 10.1186/s40064-015-1509-2 PMC4656256

[20] Monsarrat P, Kémoun P, Casteilla L, Planat-Bénard V. Broad-Spectrum Antibacterial Effects of Human Adipose-Derived Stromal Cells. Stem Cells Int. 2019. p. 5389629. 10.1155/2019/5389629 PMC6855043 31781241

[21] Yaylacı S, Kaçaroğlu D, Hürkal Ö, Ulaşlı AM. An enzyme-free technique enables the isolation of a large number of adipose-derived stem cells at the bedside. Sci Rep. 2023;13:1. 37198228 10.1038/s41598-023-34915-0 PMC10192379

[22] Shah FS, Wu X, Dietrich M, Rood J, Gimble JM. A non-enzymatic method for isolating human adipose tissue-derived stromal stem cells. Cytotherapy. 2013;15:8. 10.1016/j.jcyt.2013.04.001 23725689

[23] Oberbauer E, Steffenhagen C, Wurzer C, Gabriel C, Redl H, Wolbank S. Enzymatic and non-enzymatic isolation systems for adipose tissue-derived cells: Current state of the art. Cell Regeneration. 2015;4:1. 26435835 10.1186/s13619-015-0020-0 PMC4591586

[24] Hosseini V, Kalantary-Charvadeh A, Hasegawa K, Nazari Soltan Ahmad S, Rahbarghazi R, Mahdizadeh A, Darabi M, Totonchi M. A mechanical non-enzymatic method for isolation of mouse embryonic fibroblasts. Mol Biol Rep. 2020; 47:11. 10.1007/s11033-020-05940-3 33130988

[25] Myers JA, Curtis BS, Curtis WR. Improving accuracy of cell and chromophore concentration measurements using optical density. BMC Biophys. 2013;6:1. 24499615 10.1186/2046-1682-6-4 PMC3663833

[26] Aijaz A, Trawinski D, McKirgan S, Parekkadan B. Non-invasive cell counting of adherent, suspended and encapsulated mammalian cells using optical density. Biotechniques. 2019;68:1. 10.2144/btn-2019-0052 PMC7031820 31870165

[27] Beal J, Farny NG, Haddock-Angelli T, Selvarajah V, Baldwin GS, Buckley-Taylor R, Gershater M, Kiga D, Marken J, Sanchania V, Sison A, Workman CT, Pehlivan M, Roige BB, Aarnio T, Kivisto S, Koski J, Lehtonen L, Pezzutto D, Zhou J. Robust estimation of bacterial cell counts from optical density. Communications Biology. 2020;3:1. 33110148 10.1038/s42003-020-01371-9 PMC7591534

[28] Zhang H, Li Y, Ibáñez CF, Xie M. Perirenal adipose tissue contains a subpopulation of cold-inducible adipocytes derived from brown-to-white conversion, eLife. 2024;13:RP93151. 10.7554/eLife.93151 PMC10932542 38470102

[29] Bourgeois C, Gorwood J, Barrail-Tran A, Lagathu C, Capeau J, Desjardins D, Le Grand R, Damouche A, Béréziat V, Lambotte O. Specific Biological Features of Adipose Tissue, and Their Impact on HIV Persistence. Front Microbiol. 2019;10:2837. 10.3389/fmicb.2019.02837 PMC6927940 31921023

[30] Caputa G, Matsushita M, Sanin DE, Kabat AM, Edwards-Hicks J, Grzes KM, Pohlmeyer R, Stanczak MA, Castoldi A, Cupovic J, Forde AJ, Apostolova P, Seidl M, van Teijlingen BN, Villa M, Baixauli F, Quintana A, Hackl A, Flachsmann L, Pearce EJ. Intracellular infection and immune system cues rewire adipocytes to acquire immune function. Cell Metab. 2022;34:5. 35508110 10.1016/j.cmet.2022.04.008

[31] Zhang LJ, Guerrero-Juarez CF, Hata T, Bapat SP, Ramos R, et al. Dermal adipocytes protect against invasive *Staphylococcus aureus* skin infection. Science. 2015;347(6217):67–71. 10.1126/science.1260972 PMC4318537 25554785

[32] Teixeira L, Whitmire JK, Bourgeois C. Editorial: The role of adipose tissue and resident immune cells in infections. Front Immunol. 2024;15:1360262. 10.3389/fimmu.2024.1360262 PMC10920208 38464526

[33] Farag HS, Aly SS, Fahim KM, Fayed AA, Abdelfattah EM, El-Sayed SM, Hegazy YM, ElAshmawy WR. Management Practices of Bovine Mastitis and Milk Quality on Egyptian Dairies. Vet Sci. 2023;10:629. 37888581 10.3390/vetsci10100629 PMC10611314

[34] Kumar P, Lee JH, Beyenal H, Lee J. Fatty acids as antibiofilm and antivirulence agents. Trends Microbiol. 2020;28:9. 10.1016/j.tim.2020.03.014 32359781

[35] Shaaban MT, Ghaly MF, Fahmi SM. Antibacterial activities of hexadecanoic acid methyl ester and green-synthesized silver nanoparticles against multidrug-resistant bacteria. J Basic Microbiol. 2021;61(6):557–68. 10.1002/jobm.202100061 33871873

[36] Ansari MA, Asiri SMM, Alzohairy MA, Alomary MN, Almatroudi A, Khan FA. Biofabricated Fatty Acids-Capped Silver Nanoparticles as Potential Antibacterial, Antifungal Antibiofilm and Anticancer Agents. Pharmaceuticals. 2021;14:2. 10.3390/ph14020139 PMC7915658 33572296

[37] Galdiero E, Ricciardelli A, D'Angelo C, de Alteriis E, Maione A, Albarano L, Casillo A, Corsaro MM, Tutino ML, Parrilli E. Pentadecanoic acid against Candida albicans-Klebsiella pneumoniae biofilm: towards the development of an anti-biofilm coating to prevent polymicrobial infections, Research in Microbiology. 2021;172(7-8):103880. 10.1016/j.resmic.2021.103880 34563667

[38] Jin X, Zhou J, Richey G, Wang M, Hong SMC, Hong SH. Undecanoic Acid, Lauric Acid, and N-Tridecanoic Acid Inhibit Escherichia coli Persistence and Biofilm Formation. J Microbiol Biotechnol. 2021;28:31. 10.4014/jmb.2008.08027 PMC8513074 33046677

[39] Misra D, Ghosh NN, Mandal M et al. Anti-enteric efficacy and mode of action of tridecanoic acid methyl ester isolated from Monochoria hastata (L.) Solms leaf. Braz J Microbiol .2022;53. 10.1007/s42770-022-00696-3 PMC9151942 35149984

[40] Prasath KG, Sethupathy S, Pandian SK. Proteomic analysis uncovers the modulation of ergosterol, sphingolipid and oxidative stress pathway by myristic acid impeding biofilm and virulence in Candida albicans. J Proteomics. 2019;30:208. 10.1016/j.jprot.2019.103503 31454558

[41] Lalthanpuii PB, Lalchhandama K. Chemical profiling, antibacterial and antiparasitic studies of Imperata cylindrica. J Appl Pharmaceut Sci. 2019;9:12.

[42] Davoodbasha M, Edachery B, Nooruddin T, Lee S, Kim J. An evidence of C16 fatty acid methyl esters extracted from microalga for effective antimicrobial and antioxidant property. Microb Pathog. 2018;115:233–8. 10.1016/j.micpath.2017.12.049 29277474

[43] Kim YG, Lee JH, Lee J. Antibiofilm activities of fatty acids including myristoleic acid against Cutibacterium acnes via reduced cell hydrophobicity. Phytomedicine. 2021;91:153710. 10.1016/j.phymed.2021.153710 34461422

[44] Kapoor A, Mishra DN, Narasimhan B. Antibacterial and antifungal evaluation of synthesized 9,12-octadecadienoic acid derivatives. Pharm Lett. 2014;6:5.

[45] Nuerxiati R, Wubulikasimu A, Mukhamedov N, Mirzaakhmedov SY, Gao Y, Aisa H, Yili A. Biological Activity of Fatty Acids from Lipids of Orchis chusua. Chem Nat Compd. 2021;57:2.

[46] Rizvi SNR., Afzal S, Khan KUR, Aati HY, Rao H, Ghalloo BA, Shahzad MN, Khan DA, Esatbeyoglu T, Korma, SA. Chemical Characterisation, Antidiabetic, Antibacterial, and In Silico Studies for Different Extracts of Haloxylon stocksii (Boiss.) Benth: A Promising Halophyte. Molecules. 2023; 28:9. 10.3390/molecules28093847 PMC10180423 37175255

[47] Chowdhury SK, Dutta T, Chattopadhyay AP, et al. Isolation of antimicrobial Tridecanoic acid from Bacillus sp. LBF-01 and its potentialization through silver nanoparticles synthesis: a combined experimental and theoretical studies. J Nanostruct Chem. 2021;11:573–87.

[48] To NB, Truong VNP, Ediriweera MK, Cho SK. Effects of Combined Pentadecanoic Acid and Tamoxifen Treatment on Tamoxifen Resistance in MCF−7/SC Breast Cancer Cells. Int J Mol Sci. 2022;23(19):11340. 10.3390/ijms231911340 PMC9570034 36232636

[49] Bermúdez MA, Pereira L, Fraile C, Valerio L, Balboa MA, Balsinde J. Roles of palmitoleic acid and its positional isomers, hypogeic and sapienic acids, in inflammation metabolic diseases and cancer. Cells. 2022;11:2146. 35883589 10.3390/cells11142146 PMC9319324

[50] El-Sherbiny GM, Kalaba MH, Sharaf MH, Moghannem SA, Radwan AA, Askar AA, Abushiba MA. Biogenic synthesis of CuO-NPs as nanotherapeutics approaches to overcome multidrug-resistant *Staphylococcus **aureus* (MDRSA). Artif Cells Nanomed Biotechnol. 2022;50(1):260–74. 10.1080/21691401.2022.2126492. 36191138 10.1080/21691401.2022.2126492

